# Safe use elements of finished herbal products: insights from consumers and practitioners in Malaysia

**DOI:** 10.1186/s12906-024-04546-7

**Published:** 2024-07-03

**Authors:** Nur Syamila Mohd Roziman, Wardah Mustafa Din, Zurina Mahadi, Farida Islahudin, Mazlina Md. Said

**Affiliations:** 1 Institut Islam Hadhari, Universiti Kebangsaan Malaysia, Bangi, 43650 UKM Selangor Malaysia; 2 Pusat Pengajian Citra Universiti, Universiti Kebangsaan Malaysia, Bangi, 43650 UKM Selangor Malaysia; 3 Faculty of Pharmacy, Universiti Kebangsaan Malaysia, Jalan Raja Muda Abdul Aziz, Kuala Lumpur, 50300 Malaysia

**Keywords:** Safe use, Natural herbal products, Consumers, Practitioners, Qualitative approach, Focus group discussion

## Abstract

**Background:**

The use of finished herbal products (FHPs) among Malaysians today is expanding rapidly leading to a huge market of FHPs in the country. However, the mass production of FHPs in today’s market is alarming due to safety-use issues that could lead to serious adverse effects. Nevertheless, demands are still high for FHPs as most consumers perceived it as safe to consume as it is made from natural substances as the active ingredients. This study aims to explore the safe use elements of FHPs identified by two stakeholders: consumers and practitioners in Malaysia and further compare these elements with the current regulations.

**Methods:**

As an exploratory study, its approach is to investigate at an in-depth level of understanding of safe use elements from the involved stakeholders: consumers and practitioners. We had a total of 4 focus group discussion sessions (1 FGD session with consumer and 3 FGD sessions with practitioners) as a method of collecting data from the participants. The FGDs were conducted in local native Malaysian and then being translated by researchers without changing their meanings. Thematic analysis was done which involves methodically reading through the verbatim transcripts and consequently segmenting and coding the text into categories that highlight what the participants have discussed.

**Results:**

From the result, we found that both practitioners and consumers agreed a safe FHP must be in compliance with the guidelines from the Ministry of Health Malaysia (MOH). There are other safe use elements highlighted including halal certification, trusted over-the-counter outlets, and published reports on the safety, efficacy, and quality.

**Conclusions:**

In conclusion, both practitioners and consumers agreed that the most important safe-use element is compliance with MOH guidelines, but the depth of discussion regarding the safety elements among these stakeholders holds a very huge gap. Thus, initiatives must be planned to increase the knowledge and understanding about the MOH guidelines towards achieving a sustainable ecosystem in the safe use of FHPs.

**Supplementary Information:**

The online version contains supplementary material available at 10.1186/s12906-024-04546-7.

## Background

According to the National Pharmaceutical Regulatory Agency (NPRA), natural products are divided into five categories in Malaysia including; (i) traditional medicine, (ii) herbal remedy, (iii) finished herbal products, (iv) homeopathic medicine, and (v) natural products with therapeutic claims. In this study, the researchers are focusing on the use of finished herbal products (FHP) among consumers in Malaysia. FHP is defined as *“finished herbal products consist of herbal preparations made from one or more herbs. If more than one herb is used, the term “mixture herbal product” can also be used. Finished herbal products and mixture herbal products may contain excipients in addition to the active ingredients. However, finished products or mixture herbal products to which chemically defined active substance have been added, including synthetic compounds and/ isolated constituents from herbal materials, are not considered to be herbal.”* [1]. The use of FHPs among Malaysians today is expanding rapidly as well as the market of FHPs in the country. This is due to the high demand from consumers who consume FHPs as alternatives to treat chronic or acute diseases, and frequently used for maintaining or improving general health. The market of FHPs in Malaysia is very big and in high demand among consumers due to cultural acceptability, availability, and beliefs of its beneficial effects on overall health [2]. Furthermore, today’s blooming and rapid growth of FHPs can be associated with the marketing from the producers, sellers, and manufacturers through online marketing via the Internet [3]. However, the mass production of FHPs in today’s market has been an eye-opener, especially regarding the safety issues that could lead to serious adverse effects that could be affecting their quality of life, morbidity, and even worse, mortality [4]. Acute and chronic diseases such as renal failure and liver damage are always associated with the consumption of FHPs especially among consumers in Malaysia. This is due to some herbs being known for their toxicity and can worsen the condition of the kidney [5]. In 2023, Malaysia has received total number of five cases related to kidney failure due to the consumption of herbal products. It has involved problems such as loss of kidney function caused by high levels of creatinine and urea in blood, sudden damaged and injured kidney, and deterioration of pre-existing renal issues [6].

There are many factors leading to adverse effects caused by FHPs such as herb-drug interactions, toxic chemicals or heavy metals, poor manufacturing and regulatory processes from manufacturers, and adulteration [3]. Adulteration in natural products is often seen as common issue that can caused adverse reactions to the consumers. Adulteration happens when the producers mix the product ingredients with hazardous compounds which is usually undeclared until the consumers experience unwanted adverse effects. Hence, the government has urged all consumers to re-check the registration number of any FHPs before buying it from the market [7]. This is due to the registration requirement and safety assessments of FHPs being less stringent compared to synthetic pharmaceuticals [3, 7, 8]. The concern multiplies as there are regulatory gaps whereby herbal products can be registered as food under the Food and Drug Administration (FDA) rather than as medicine under National Pharmaceutical Regulatory Agency (NPRA). All medicine including herbals must be registered and follow the DRGD guidelines according to the category of the medicines. As for finished herbal products, NPRA has provided a detailed registration guideline to follow which is the Drug Registration Guideline Document (DRGD). In this study, we referred to the DRGD Third Edition, Fourth Revision January 2023, Appendix 7. Recently, NPRA also has introduced Guideline of Natural Product with Modern Claim which was released in April 2024.

The main issue with herbal-based products in Malaysia is many manufacturers will classify their products as food supplements which will not be a requirement to register the products as FHPs which is more obligated to follow the regulations under the authority body, NPRA [3]. In the same vein, many producers and online agents who do aggressive marketing always convince potential consumers by promising positive effects on their health and that the product is effective to cure certain diseases. However, many of these poor-quality products are mostly not even monitored by the authority because most of them are not registered under NPRA hence the easily trusted consumers became the victim of those irresponsible sellers [5].

Many factors have been contributing to the increase in the consumption of FHPs such as their accessibility and affordability [2]. These products are easily obtained by consumers through online or physical platforms as they are non-prescription and over-the-counter products in the market [3]. As FHPs may contain many active ingredients in the formulation and can bring effects to the body, it is safe for any consumers to be alert of any adverse effects and refer to healthcare professionals before consuming any FHPs from the market. At the consumer level, they still lack knowledge and awareness regarding the safe use of FHPs [5]. Studies show that the non-disclosure intake of FHPs among the public is high. This is because they feel it is not important to report or discuss with the practitioners about their consumption of FHPs and the fear of rejection from practitioners [9]. It is important to get approval from healthcare providers as they know better about your health status, especially those who are taking any prescribed medications. This is to avoid any herb-drug interaction that can cause serious adverse effects. Consumers should have a profound level of awareness of the safe use of FHPs to prevent the seriousness of the adverse effect rooting from consuming any FHPs.

As practitioners, they are responsible for providing advice and assessing the patients who will seek guidance before consuming any FHPs [10]. As the main source of information for the patients’ use of FHPs, professional practitioners are responsible for advocating the patients to carefully choose any FHPs in the market since consuming FHPs are non-prescription drugs that can be obtained from any premises in the market nowadays [11]. Since many of the patients are reluctant to tell their healthcare professional about taking FHPs, practitioners rarely were asked by the patients about FHPs during consultation. This may lead to serious herb-drug interaction and other adverse events that could be stopped if this matter were taken seriously by both stakeholders [12].

Nevertheless, there is still demand from consumers as most have perceived these FHPs are safe to consume as they are made from natural substances such as parts of plants as active components and effective as well. When there is demand, FHPs will always be widely available in the market as over-the-counter products on most premises [11, 13]. Other than that, the public still has access to FHPs from online purchasing platforms and social media [7]. It can be summarised that the safe use of FHPs is very crucial in ensuring healthy lives and promoting well-being for all ages. This list of safe-use elements by consumers and practitioners is pivotal in developing policies for the safe use of FHPs and ensuring effective initiatives are being taken regarding this issue to bring toward a healthy society. The aim of this study is to elucidate the safe use elements of FHPs in Malaysia among two stakeholders which are the consumers and practitioners.

## Research methodology and data collection method

This research is an exploratory study which is conducted using a qualitative methodological approach. The main research question mainly highlighted how these stakeholders would define the safe use elements of finished herbal products. As an exploratory study, its approach is to investigate at an in-depth level of understanding of safe use elements from the two stakeholders including consumers and practitioners in Malaysia. We had a total of 4 focus group discussion sessions (1 FGD session with consumer and 3 FGD sessions with practitioners) involving 17 participants as a method of collecting data (Demographic data as attached in Appendix 1). All FGDs were held in Kuala Lumpur which is a state in Peninsular Malaysia and we have organised the FGDs in separate sessions to ensure the input of information we got from the discussion is based on their expertise. The FGDs were conducted in local native Malaysian and translated by researchers without changing the meanings.

### Recruitment and participants

In the early process of recruitment for consumers, the researchers blasted the invitation through social media such as Facebook and WhatsApp in May 2022. The targeted group of our focus groups was consumers and practitioners in Malaysia. The criteria for the consumers includes they must be Malaysian citizens, specifically those who are consuming any finished herbal products in the market for at least one product for the last one year. As for practitioners, they must have experience in advising patients and spreading awareness about safe use of finished herbal products. We have divided the practitioners into three sessions of FGD according to their roles as medical doctors and dentists, pharmacists, and nurses. Their insights on the safe use elements of finished herbal products are slightly different as their experience in treating patients are different. However, during data analysis, we coded the safe use elements accordingly to build the themes of safe use elements of finished herbal products through a thematic analysis process.

### Focus group procedure

Before FGD was conducted, the participant had been informed in advance regarding the purpose of the study and research objectives were highlighted. This is to ensure they have a clear understanding of the research topic and research objectives. All participants have agreed to be a part of the FGD and have given informed consent. A semi-structured protocol was prepared to guide the discussion during the FGD. It was an open-ended discussion where the participants were able to discuss and share their opinions openly without limitation. However, the researchers have prepared structured questions for every FG to facilitate the same question to the participants. Each FGD took approximately two hours to complete the discussion and was moderated by 3 researchers. The discussion was recorded using an audio recorder and a video recorder to make sure the information gained was clearly captured to be easily transcribed later for data analysis by researchers. Participants have been informed earlier and agreed to be recorded for research purposes only. After each session of FG, it is important for the researchers to determine if the information gained from the discussion is exhaustive and saturated. If the data is exhaustive, the researchers can proceed to another FGD with the next group and move along with thematic analysis using existing data. Exhaustive data is determined when the stakeholders have successfully listed all elements of safe use similarly when the moderator is querying them with the questions from the FGD protocol prepared by the researchers. For the FGD with consumers, extra questions were asked by the moderator on what products were being consumed and what active herbal ingredients were in each product to the consumers.

### Saturation and exhaustive data

Data saturation is to measure the adequacy of the safe use elements given by the stakeholders. Our method of data collection from a series of FGDs with consumers and practitioners needs to be measured based on the saturation of the data. Thus, reaching data saturation is a critical component in order to validate our data in this study [14]. To measure the data saturation from the series of FGDs, it can be referred to the discussion on theoretical saturation introduced by Glaser and Strauss (1967: p. 61). This theory mentioned the term data saturation which means there are no new findings to address the research question. Furthermore, from the data gained from the discussion, researchers can create a different set of categories from the original data. Probing questions by the moderator is important as it is to identify if the research questions have been properly answered and make sure there is no new information being generated from the probing questions [15].

## Data analysis

After the data collection process ends, the audio that was recorded is later transcribed and thematic content analysis was done aided by a qualitative data analysis software, ATLAS.ti version 23.2.0 [16]. The FGDs were conducted until the elements mentioned by these stakeholders were saturated and exhaustive enough to make it a list of the safe-use elements in a model concept. The theme-building process is to outline the themes of safe use elements by the stakeholders from the series of FGDs conducted before. In determining the themes, inductive analysis was done where the analysts need to identify the themes derived from the data. Analysing the transcripts of consumers and practitioners from the discussion to figure out the themes of safe use is crucial in order to produce a model concept of safe use elements of FHPs. To confirm if the data is exhaustive, we can identify it when all stakeholders have listed the same elements during the FGDs. Each descriptive code will be glued together as a theme that is not limited to only one theme but more than one. Elucidating safe use elements of herbal products by consumers and researchers is the main objective of this research study. To create a theme from the descriptive code, we have followed the steps introduced by Braun and Clarke in the thematic analysis method which initially one must have completed and familiarised with the data to generate codes. From there, the themes can be determined through a set of codes that must be reviewed later to make sure it does not overlap with other themes. After finalising the code of each theme, reviewing must be done to ensure that the code that has been listed in the theme matches and is sorted accordingly under the right theme. To organise the occurring themes, the researcher can put them in an idiographic way such as through tables, mind maps, and theme trees to show the linkage between every theme [17]. Then, researchers discuss the themes with regulators through a round table discussion to figure out the main themes that are best to be listed in the framework for safe-use natural herbal products.

## Results

### Herbal ingredients contained in the FHPs mentioned by consumers

One of the main criteria for participants in FGD with consumers, is that the participants must at least consume herbal products once before. Hence, we share the herbal ingredients contained in the FHPs that are being mentioned by participants throughout the FGD as mentioned in Table 1 below.

**Table 1:** Name of herbal ingredients mentioned

No.	Name of herbal ingredients mentioned	Scientific name
1.	Habbatus sauda	*Nigella sativa*
2.	Olive Oil	*Olea europaea L.*
3.	Pomegranate	*Punica granatum L.*
4.	Virgin Coconut Oil	*Cocos nucifera L.*
5.	Bitter gourd	*Momordica charantia L.*
6.	Apricot seeds	*Prunus armeniaca L.*
7.	Mixed herbal capsules	-
8.	Mixed herbal drinks	-

### Safe use elements of herbal products

There are five safe use elements of FHPs identified by both stakeholders. Furthermore, safe use elements agreed upon only by one stakeholder are also discussed in this article. Among the list of safe use elements discussed by both stakeholders, only one safe use element is aligned with the current regulation from MOH Malaysia which is a safe FHP must comply with the MOH guidelines. Under this element, there are occurring sub-elements that follow the list from the guidelines from MOH Malaysia. It is identified that these elements coincide with the guidelines disseminated by the regulators, but the depth of safety elements among these stakeholders holds a very huge gap to achieving a sustainable ecosystem in the safe use of FHPs.

Based on the findings, both stakeholders have listed several elements of the safe use of FHPs. The safe use elements of FHP mentioned by the stakeholders cover: (i) in compliance with the guidelines from the Ministry of Health Malaysia (MOH), (ii) halal certification, (iii) trusted over-the-counter outlet, (iv) established brand manufacturers, and (v) published report on safety, efficacy, and quality. There are also other occurring elements that have been discussed among these two stakeholders to strengthen their point of view for the safe use of FHPs depending on their role. Even though MOH has prepared a thorough guideline on its official website, nonetheless there is still a huge gap of awareness among these stakeholders, especially the consumers when they are still not able to capture the themes of safe use elements of FHPs from the existing guidelines. This has shown that the understanding and awareness of safe use of FHPs are different between stakeholders. There are gaps in the safe use for each stakeholder that must be filled in by creating awareness for everyone on this matter.

#### Safe use elements agreed by consumers and practitioners

##### In compliance with the guidelines from the Ministry of Health Malaysia (MOH)

Both stakeholders’ most frequently mentioned element for the safe use of FHPs is the FHP must be following the guidelines from MOH Malaysia. Under this element, only one safe use element is aligned with the current regulation from MOH Malaysia which is a safe FHP must comply with the MOH guidelines. There are six sub-elements emerged during FGDs that are also mentioned in the current regulation of MOH including i) registration number, ii) security label hologram, iii) dosage information, iv) GMP label, v) detailed label and packaging, and iv) list of ingredients. Some of these sub-elements can be found in the natural product registration guidelines Malaysia, Drug Registration Guideline Document (DRGD) Third Edition, Fourth Revision January 2023, Appendix 7. As for GMP label, it only applies to the manufacturers who need to apply for GMP status for the factory, however, in the DRGD document, the GMP label is prohibited on product label because certification renewal is on a yearly basis [1].

*“Firstly, I check if the product is MOH registered.”* (C04).

*“It must be registered to MOH.”* (P04).

*“So, the safety scope in terms of enforcement, we will check if the product is registered or not.”* (P05).

*“…and search for the registration number on the website checker to see whether it is registered or not”* (C05).

*“In terms of enforcement, the two things, MAL number with the end of T, protection product, and hologram label”* (P04).

*“…After that, when we check the packaging of the product, it has a halal certification label, serial number from MOH.”* (C05).

A complete label needs to be included on the packaging including disclaimer, indication, and contraindications that can easily be referred to with simpler terms that can be understood by the consumers and practitioners.

*“To me, if the product contains a disclaimer for example, it says if symptoms persist consult your doctor or please stop consuming, I feel safer about the product”* (C05).

*“…. What is its use for us? Why do we take it? For instance, cat’s whiskers are one of the herb’s use to cure high blood pressure. But what is the right dose? If it’s in tea form, we must use the safe dose as it differs to those people without hypertension and with.”* (P05).

Other than that, the FHPs must include the dosage information for daily intake and the consumer must follow the dose suggested on the label.

*“Usually on the packaging, it is complete. I mean about dosing, and then the ingredients, they will declare it”* (P02).

*” Do not consume it drastically, if on the bottle mentioned take two, then two.”* (P01)

List of ingredients must be presented on the packaging as it is one of the guidelines listed by MOH for a safe product whereas, GMP logo is optional. Although GMP is not permitted to be presented on the packaging of the FHPs by NPRA, both stakeholders did mention GMP logo as one of the safe use elements that must have in a FHP before consuming. GMP is part of quality assurance from acquiring raw materials until distributing the finished products to the market which ensures products are consistently produced and monitored according to the quality standards required to fulfill their intended use [18]. Manufacturers have the option to apply for GMP certification to produce their products. Overall, the GMP label is one of the important safety aspects to ensure the quality and nature of FHPs are well maintained.

*“Must have GMP logo”* (P08)

*“. and then check if it follows GMP or not.”* (P06)

*“I mean when I say it is safe, we must be aware of the ingredients… We have to check especially on the bottle, has been capsulated, we need to check the ingredients.”* (P01)

*“To me, ingredients are important even though all original products must list the ingredients on the packaging of the box. I haven’t found any products that come with a box that did not mention the ingredients, it only has a name and not the ingredients, no, it must have (ingredients).”* (C07)

Registered FHPs are the most important safe use elements agreed upon by both stakeholders. Also, other requirements listed by MOH must be available upon checking the product via online or in-store to verify if the product is MOH approved or not.

##### Halal certified

Based on the finding, we can conclude that halal-certified products are important to consumers as they feel more confident in consuming these halal-certified products compared to other products without halal certification. They always check for the halal certification logo on the packaging of the product before deciding to purchase the product. As for practitioners, they also agree on the importance of halal certification as it can be said that the ingredients used to prepare FHPs is halal, especially for Muslim consumers.

*“Usually it has halal logo”* (C01)

*“Other than halal…”* (CO2)

*“… after that, I will check the halal symbol first”* (CO4)

*“Then, when we look at the product box, there’s halal logo.”* (C05)

*“Before getting the halal logo, it must have MAL number. Means, before applying for halal, the product must get MAL number. Then only can go for halal logo. Since the product is halal-certified, it has added value on the product of its quality, safety, and efficacy. And also halal is a added value to the meaning of safe in syarak. When NPRA confirmed a product is safe, effective, and quality, it is safe or in syarak for Islam teaching, it can be consumed.”* (P05)

*“Yes, it is important (halal logo)”* (P07 & P08)

*“To me it is important. We need to know the ingredients used are from halal resources.”* (P07)

*“Since we don’t know what other extracts were put in, so it needs to have it (halal logo)”* (P08)

As for halal status on FHPs, both stakeholders feel it is as other additional values of safe use of FHP.

##### Trusted over-the-counter outlets

FHPs are self-prescription medicine that can be accessed on many premises in the market. Based on the discussion with both consumers and practitioners, they mentioned that FHPs which are sold in pharmacies are safer to buy rather than any FHPs sold through online platforms such as Facebook or small outlets or in beauty centres or small stalls. This is due to the presence of healthcare professionals in the pharmacies that can be referred to when the consumers are looking for the best option of FHPs that is suitable for their needs whether to treat or prevent diseases. Other than that, FHPs sold in the pharmacies are mostly MOH-certified which usually follows all the standards regulated by MOH for the safety, efficacy, and quality of the products. This is the main issue with online selling that could lead to serious adverse effects for most consumers nowadays.

*“Secondly, when I’m buying a product, the place of the product sold will be my concern. If it is from a pharmacy, then it is one of the factors that give confidence to me”* (C07)

*“Means if it is sold from a pharmacy, then it is sure that all products in the pharmacy are safe. If we were to compare those products sold from “jamu shop”, not all products are guaranteed to be safe. But if it’s a product sold in a pharmacy, in sha Allah everything is safe. It is counted as the first line for safety measures.”* (P04)

From this finding, even though FHPs can be purchased at many premises as over-the-counter products, pharmacy is the most common premise to get safe FHPs agreed by consumers as there are pharmacists as the practitioner that are able to guide them when they are purchasing FHPs. As for the practitioners, they also encouraged the consumers to always seek advice from professional practitioners in consuming and purchasing FHPs.

##### Established brand manufacturers

With many FHP manufacturers in Malaysia, both stakeholders agreed that the reputation and the brand of the manufacturer is one of the safe use elements to ensure the safety of herbal products available in the market. For consumers, they feel more secure by searching the background of the producer before purchasing the herbal product as they will only buy a product that is safe and genuine to them.

*“Also, when I did my study on the company and it showed the location of the company in Penang and the factory is well-established, I feel secure to consume the product”* (C05)

*“… I mean when I mentioned the product and people surrounding are familiar with the brand and the producer.”* (C07)

*“There is also the company such as Shaklee or Herbalife, the famous ones so I feel more confident about it.”* (P02)

Well-known product branding or producers are more favoured by both stakeholders to define the safe use of FHPs. From background checking, big-name companies usually bring positive attitudes which could lead to purchasing decisions. This has created the concept of brand trust as for both stakeholders, well-known producers are more reliable and trusted in the safety of the herbal product. The concept of brand trust here shapes the meaning of confidence for consumers in the herbal product to the product’s intended purpose [19].

##### Published report on safety, efficacy, and quality

Other than following the specified requirements to determine the quality and safety of FHPs, it is also important for the product to have additional scientific support proven by empirical data through various tests. These tests usually identify the presence of heavy metals and microbial contamination if found in the product. This can prevent any adverse effects, especially for chronic patients.

“*So, I will feel more confident to consume since the product has undergone tests rather than just simply spreading words without any evidence”* (C05)

*“To me, it is safe when a product is tested. Meaning, when a product is produced with evidence supports and research, not only one piece of evidence but more than one, it is better. At least it is clinically tested, that is the best.”* (P05)

Based on the discussion with the practitioners, they agreed on the statement of when a FHP is said to have been tested, the test is only for some active ingredients used in the product. It is not tested as a finished product. This holds a gap in safety elements on tested herbal products in the market.

## Safe use elements agreed upon by either consumers or practitioners

This study has other findings on the safe use elements of FHPs agreed upon by only one stakeholder (Refer to Table 2). Other safe-use elements listed by consumers that are not mentioned by practitioners comprise of (i) testimonials, (ii) self-perception of efficacy, (iii) product’s ambassador, (iv) popular product (virality), (v) the use of “natural” term, and (vi) herbs as sunnah diet. After must be in compliance with guidelines from MOH Malaysia, testimonials are the second most mentioned safe use element of FHPs agreed upon by consumers during the FGD.

On the other hand, practitioners do not agree especially with testimonials from other consumers as safe use element. Moreover, there are other additional safe use elements listed by the practitioners who are in the early process of preparing a FHP, it is suggested to refer to the Malaysian Herbal Monograph (MHM) report to ensure the herbs used in the making of FHP are beneficial and safe to be consumed by humans. The MHM document is crucial in determining proper indication and comprehensive botanical information on any herbs used to produce FHPs since the main ingredient is based on herbs [20].

**Table 2:** Safe use elements agreed upon by either consumers or practitioners

Safe-use elements/Stakeholders	Consumers	Practitioners
Testimonials	✔	
Perception of efficacy	✔	
Popular product	✔	
“Natural” term	✔	
Herbs as sunnah diet	✔	
Refer to the Malaysian Herbal Monograph report		✔

### Safe use elements agreed upon by consumers only

#### Testimonials

From the discussion, these consumers have been depending on other consumers’ testimonies on their experience with the FHP they are interested in. Before deciding to make a purchase of the FHP, especially through online selling platforms, these consumers will make sure to do some reading of testimonials from other consumers.

*“Firstly, I will look up testimonials from other consumers. The more testimonies, the higher the chance I will proceed to purchase the FHP. This is because I feel more convinced that the product is safe and proceed to consume by reading the positive testimonies from other consumers”* (C07)

*“Yes (testimonials from other consumers). When I need to inquire more about the product, the online seller will contact me directly and guide me to find the most suitable product based on my health status. Testimonials from other consumers were also provided when asked…”* (C04)

*“If I was looking for a FHP, I will read the testimonials in the comment section on Facebook. From there, I gained information regarding the FHP from other consumers. They usually will leave honest reviews. If there was a negative testimony, I will contact them to ask more about the product. Usually, they will explain more, then only I will decide whether to proceed with purchasing the product or not. If there were a lot of negative feedback, I will not buy the product. Normally I will query other consumers about the product just to ensure the product is safe to consume.”* (C03)

According to Berger and Calabrese’s (1975) uncertainty reduction theory in the online shopping context, when a shopper is uncertain about a certain product, they will turn to the seller or other shoppers to ensure the product will meet their expectations and perform its intended function. When the shopper has gained the right amount of information about the product, the uncertainty will be reduced which could influence the shopper to purchase the product [21]. From this theory, we can learn these consumers prefer testimonials from other consumers to reduce their uncertainty towards the targeted FHP.

#### Self-perception on efficacy

Every FHPs have its intended function to perform which is expected by every consumer. From this study, the consumers perceived an effective FHP as one of the safe use elements. They agreed on the effectiveness of the FHP depending on the positive reaction to their overall health and did not cause any negative effects upon consuming the FHP.

*“I’d know if the product is effective when it gives positive effect to my body”* (C07)

*“Another thing, when I was using the product, I can feel my body was more energetic*and lighter. Even my movements were faster, and I had good nights of sleep.” (C04)

*“From the plant species, I would look up the benefits and functions on the Internet. When I read more about it, I’d make sure the product’s indication aligned with the function of the plant. Then only I feel safer to consume the FHP”* (C05)

Effectiveness was also determined from the period of time after consuming the FHP. They prefer FHP which can give immediate effect based on the indication of the product to their body. If the product fails to perform based on its indication in a certain period, they will stop consuming the FHP and replace it with a new FHP with the same function.

#### The use of the “natural” term

This perception is common among consumers on the “natural” term that is being emphasised on the label or packaging of the product. When the “natural” term is highlighted on the product, they have identified it as a safe herbal product that only consists of natural substances that they considered harmless to their overall health.

*“They only use natural ingredients, and it is not mixtures”* (C07)

*“From the ingredients, it stated fruits are the main active element of the product. We can eat the fruit after boiling it. It made me feel safe to consume the FHP.”* (C04)

For these consumers, it is important for them to confirm the FHPs are made from natural ingredients based on the packaging of the product. The “natural” term highlighted on the packaging is useful to attract the consumer to buy the FHP.

#### Herbs as sunnah diet

This element was mentioned by Muslim consumers during the FGD. Sunnah diet is one of the many teachings by Prophet Muhammad (p.b.u.h) that brings benefits to humans’ general health and overall life.

*“I prefer sunnah diet product. I feel safe about it”* (C02)

*“Since the Al-Quran has stated habattus sauda and olive are one of the sunnah diet, I have been consuming FHPs from these sunnah diets and I feel safe about it”* (C01)

*“The main ingredients were raisins and other sunnah diet…”* (C04)

The consumers trust in the safety and quality of herbs that are recognized as sunnah food used in the FHPs. These sunnah foods are proven beneficial and nutritious to human bodies. Islamic teaching in food intake is very straightforward when Islam has taught to only consume beneficial and quality food to ensure good health towards a better lifestyle. Every food consumed will determine the individual’s personality and well-being. Thus, it is important for every Muslim to consume safe and quality food which includes sunnah food [22].

#### Popular product

A well-known product is perceived as a safe FHP by the participants. It is important for these consumers to get recommendations from other consumers of the targeted FHP before purchasing it.

*“Many other consumers have been consuming the same FHP… I was told that the product is good by other users… Testimonies from other consumers made it a more preferable product than other FHPs from the market”* (C07)

Other than purchasing a FHP from an established brand manufacturer, they also find it necessary to purchase FHP that is familiar to other consumers. The reviews were made through online selling platforms and social media where they can communicate directly with other consumers to seek information about the targeted FHP.

### Safe use elements agreed upon by practitioners only

#### Refer to the Malaysian Herbal Monograph report

The herbal Monograph report contains collected knowledge of specifications and indications of the raw materials that are useful as guidance to produce a safe, efficacy, and quality FHP [23].

*“…since we have the herbal monograph, we can refer to it”* (P05)

From the report, every plant species will be discussed further based on the characteristics with supporting evidence that is important to ensure any raw materials used in the production of a FHP is safe to be consumed by the consumers.

## Discussion

To our knowledge, this is the first study conducted in Malaysia that discussed the safe use elements of FHPs elicited by consumers and practitioners as stakeholders in the natural health products ecosystem. Most studies on natural health products in Malaysia usually focused more on the factors associated with natural health product use among consumers [2, 11, 13]. The discussion on safety and quality of FHPs are rarely discussed in much research, especially in Malaysia. There are two stakeholders involved in this research namely consumers and practitioners in effort to acquire representative and fair views on safe use elements of FHPs in Malaysia. In this article, consumers and practitioners point of view on safe use elements will be discussed and compared to learn the discrepancies in their perception and awareness. There are three official documents from MOH Malaysia that were used as the main references in this study. These documents are natural product registration guidelines from the Ministry of Health Malaysia (MOH), DRGD Third Edition, Fourth Revision January 2023, Appendix 7 and 33, and the Product Advertisement Approval Guide. Safe use frameworks from other countries and international bodies also were used to retrieve the elements that have been established elsewhere. This will allow measures to be taken to fill in the gap and to increase the awareness on safe use elements of FHPs.

In this study, we have learned the safe use elements of NHPs of the consumers and practitioners (Table 1). It is also evident that some safe use elements were agreed upon by both the consumers and the practitioners, while some were not. Most of the elements are in line with the guidelines disseminated by the regulators however, the depth of the understanding regarding all the safe-use elements differs between the consumers and the practitioners. The participants have also suggested good practices in regard to NHPs consumption including (1) health status monitoring before and during consumption and (2) be well-informed before taking any natural health products. These gestures highlighted the concern of the participants about their health and safety.

Based on the result, both stakeholders agreed that the main safe use element of FHPs is compliance to MOH guidelines. The guideline is regulated by National Pharmaceutical Regulatory Agency (NPRA) under the natural products category known as Drug Registration Guidance Document (DRGD) as reference. This document will be revised accordingly to any new scientific findings that can be accessed on their official website. As for this research, we are referring to the DRGD third edition, fourth revision January 2023 Appendix 7 document as a reference guide to elaborate the point on the first safe use element discussed by the stakeholders [1]. DRGD is a document published by NPRA as the secretariat to the Drug Control Authority (DCA) to regulate the registration and licensing scheme and post-registration activities for pharmaceuticals and cosmetics that shall follow the Control of Drugs and Cosmetics Regulations (CDCR) 1984 [8]. Registered products are obviously safer to be consumed compared to those unregistered and untested products. Thus, this can lead to a serious concern for the government and health authorities to tackle this issue to ensure the public is safe from these types of unregistered products. It has been observed that many of these products are foods or dietary supplements in Malaysia as such, evidence of quality, efficacy, and safety of these herbal products is not required before marketing it into the market [8]. In other words, these products are not obliged to CDCR therefore its safety is questionable.

In terms of awareness and education initiatives about the safe use of medicines, the government of Malaysia has introduced a few alternatives especially for the consumers to be more aware of their medicine. This includes a website and an application to check their product by scanning or inserting the MAL number on the product to check if the product is genuine and registered to MOH. Other than that, they will hang posters for awareness and educational purposes, mostly in healthcare facilities such as health clinics, hospitals, and pharmacies. There is also a national-level program introduced by MOH called ‘Know Your Medicine Programme’ or *‘Kenali Ubat Anda’* that should be implemented in healthcare facilities, universities, and schools in a way to spread awareness about the safe, quality, and effective use of FHPs. It emphasised the 5Rs concept for this program which includes (i) the right patient, (ii) the right medicines, (iii) the right dose, (iv) the right route of administration, and (v) the right time of administration. However, this study shows that the communications between the patients and the practitioners regarding patients’ FHPs intake are very minimal which require attention from both parties. Patients should understand the importance of consulting or informing the practitioners who attended their case of their intention to take or currently taking FHPs. The practitioners on the other hand, should always be able to advise other than being supportive and understanding of the patients interest in FHPs. Therefore, practitioners need to be adequately knowledgeable of FHPs to qualify them for giving advise of FHPs to their patients. Findings from FGDs with practitioners however indicate that most of the practitioners are not familiar with the documents on FHPs which they can refer to. Thus, they only advise their patients with their own understanding and limited knowledge about natural products. Most practitioners and consumers also did not know that they can and should make a report on the NPRA website in regard to the adverse effects caused by FHPs [5, 9].

To increase the awareness level among the stakeholders, it is important to create a better approach from the top down to ensure the quality of life, especially at the consumer level. One of the alternatives that should have been suggested by this research was to provide a framework of safe-use elements of NHP that can be referred to by all stakeholders including practitioners and consumers [24–27]. Alongside the safe use elements of FHPs from the study, there are also additional good practices that have been mentioned such as health status monitoring pre and post-consumption and one must be well-informed before taking any natural health products, suggested by both practitioners and consumers [28]. It is identified that these elements coincide with the guidelines disseminated by the regulators, but the depth of understanding regarding the safety elements among these stakeholders holds a very huge gap to achieving a sustainable ecosystem in safe use of NHPs [29]. It can be summarised that the safe use of natural health products is very crucial in ensuring healthy lives and promoting well-being for all ages. This list of safe-use elements by consumers and practitioners is pivotal in developing policies for the safe use of FHPs and ensuring effective initiatives are being taken regarding this issue to bring toward a healthy society and environment.

This paper has some limitations that need to be discovered in other future research. Since this is a qualitative and exploratory study, the level of understanding of the safe use concept, is yet to be determined. Furthermore, considering that this research only obtained the safe use elements through a small number of participants, it may not be reflective of the perspective of all consumers and practitioners in Malaysia. Nevertheless, the participants for the FGDs were purposefully chosen to elaborate on the safe use elements to create a concept model that can later evolve into a complete model of safe use elements that can be referred to all. To strengthen and discuss more on the safe use elements of FHPs listed in this study, future researchers can conduct a quantitative study with a high number of participants with the hope of establishing it as a vital tool to bridge the gap among all stakeholders in Malaysia.

## Conclusions

In this article, we are elucidating the safe use elements of FHPs through exploratory study and a series of focus group discussions with consumers and practitioners in Malaysia. There are five safe use elements of FHPs elucidated by both stakeholders. Based on this study, the themes of safe use elements of FHPs must follow official guidelines from MOH, halal-certified, validated premises, good reputation of the manufacturers or producers, and the evidence-based product has been obtained from the FGDs with the stakeholders. FHPs should be properly regulated and licensed to warrant its safety. Furthermore, safe use elements agreed upon only by one stakeholder is also being discussed in this study. Among the list of safe use elements discussed by both stakeholders, only one safe use element is aligned with the current regulation from MOH Malaysia which is that a safe FHP must comply with the MOH guidelines. Under this element, there are occurring sub-elements that follow the list from the guidelines from MOH Malaysia. It is identified that these elements coincide with the guidelines disseminated by the regulators, but the depth of understanding regarding the safety elements among these stakeholders holds a very huge gap to achieving a sustainable ecosystem in the safe use of FHPs.

We hope from this research, the policymaker can pay more attention to these safe elements to execute more plans in the future that could bring benefits especially for consumers to practise the safe use of FHPs and increase more quality FHPs in Malaysia. It can be summarised that the safe use of FHPs is very crucial in ensuring healthy lives and promoting well-being for all ages. This list of safe-use elements by consumers and practitioners is pivotal in developing policies for the safe use of FHPs and ensuring effective initiatives are being taken regarding this issue to bring about a healthy society and environment.

### Electronic supplementary material

Below is the link to the electronic supplementary material

Supplementary Material 1

## Data Availability

The datasets used and/or analysed during the current study are available from the corresponding author upon reasonable request.

## Abbreviations

FHP: Finished herbal product
MOH: Ministry of Health
NPRA: National Pharmaceutical Regulatory Agency
FGD: Focus group discussion
DRGD: Drug Registration Guidance Document
