# Supplementary material for: Safe use elements of finished herbal products: insights from consumers and practitioners in Malaysia
Source: BMC Complement Med Ther. 2024 Jul 3;24:252. doi: 10.1186/s12906-024-04546-7 (PMC11223321; doi:10.1186/s12906-024-04546-7)
Supplement: Supplementary file 1 — Supplementary Material 1 [file 12906_2024_4546_MOESM1_ESM.docx]

Appendix 1: Demographic profile of FGD participants.

| **Stakeholders** | **FGD Session** | **Participant code** | **Demographic profile of participants** |
| --- | --- | --- | --- |
| Consumer | 1^st^ | C01 | Male, adult, businessman, Selangor |
|  |  | C02 | Female, youth, student, Selangor |
|  |  | C03 | Female, youth, student, Selangor |
|  |  | C04 | Female, adult, teacher, Selangor |
|  |  | C05 | Female, youth, bank officer, Kuala Lumpur |
|  |  | C06 | Male, youth, insurance agent, Kuala Lumpur |
|  |  | C07 | Male, adult, army officer, Kuala Lumpur |
|  |  | C08 | Male, adult, IT worker, Kuala Lumpur |
| Practitioner | 1^st^ | P01 | Female, medical doctor, private clinic |
|  |  | P02 | Female, dentist, government clinic |
|  |  | P03 | Female, medical doctor, government clinic |
|  | 2^nd^ | P04 | Female, pharmacist, government pharmacy |
|  |  | P05 | Male, pharmacist, community pharmacy |
|  |  | P06 | Male, pharmacist, community pharmacy |
|  |  | P07 | Male. Pharmacist, community pharmacy |
|  | 3^rd^ | P08 | Female, nurse, government hospital |
|  |  | P09 | Female, nurse, government hospital |
